# Heredity

**Published:** 1913-01

**Authors:** 


HEREDITY. So much has been written about the contrast between the descendants of Jukes, the criminal, and Jonathan Edwards, the respectable Yankee, that we reproduce the following table from the *Medical Times*. After all it is questionable how much is heredity and how much environment.

Max Jukes.
(Born 1720.)

1200 descendants identified.
300 in the poorhouse.
300 died in childhood.
440 viciously diseased.
400 physical wrecks.
50 notorious prostitutes.
7 murderers.
60 habitual thieves; averaged twelve years in jail.

Jonathan Edwards.
(Born 1703.)

1394 descendants identified.
295 college graduates.
1 Vice-President.
3 U. S. Senators.
12 college presidents.
65 college professors.
60 physicians.
100 clergymen.
75 army and navy officers.
60 prominent authors.
100 lawyers.
80 public office holders.

Recently, we have seen a similar, though less detailed abstract between the illegitimate and the legitimate descendents of a Revolutionary soldier. But in the genealogy of the G. Family is mentioned an illegitimate branch in which the G. ancestor was selected from three possible propositi because the baby was thought to resemble him more than the others. Yet the genealogy ascribes fully as high a respectability to this putative branch as to the legitimate family.

